# Pharmacokinetics of Hyperthermic Intrathoracic Chemotherapy following Pleurectomy and Decortication

**DOI:** 10.1155/2012/471205

**Published:** 2012-04-05

**Authors:** Paul H. Sugarbaker, O. Anthony Stuart, Christopher Eger

**Affiliations:** ^1^Washington Cancer Institute, MedStar Washington Hospital Center, Washington, DC 20010, USA; ^2^Thoracic Oncology Center, MedStar Washington Hospital Center, Washington, DC 20010, USA

## Abstract

In patients with pseudomyxoma peritonei or peritoneal mesothelioma, direct extension of disease through the hemidiaphragm may result in an isolated progression of tumor within the pleural space. We monitored the intrapleural and plasma levels of mitomycin C and doxorubicin by HPLC assay in order to determine the pharmacokinetic behavior of this intracavitary use of chemotherapy. Our results showed a persistent high concentration of intrapleural drug as compared to plasma concentrations. The increased exposure for mitomycin C was 96, and the increased exposure for doxorubicin was 241. When the clearance of chemotherapy from the thoracic cavity was compared to clearance from the abdomen and pelvis, there was a considerably more rapid clearance from the abdomen as compared to the thorax. The pharmacologic study of intrapleural chemotherapy in these patients provides a strong pharmacologic rationale for regional chemotherapy in this group of patients.

## 1. Introduction

In a majority of patients, the instillation of chemotherapy into the pleural space is a palliative treatment designed to reduce or eliminate debilitating accumulations of peritoneal fluid. In these patients, disease outside of the pleural space precludes any reasonable attempt to definitively resect the cancer within the pleural space. An exception to this is pleural mesothelioma, where the thoracic surgeon performs a pleurectomy and decortication of the lung in an attempt to achieve long-term survival in patients with a limited extent of pleural mesothelioma. Also, some diseases may progress by direct extension through the hemidiaphragm and involve the thoracic cavity. If disease control within the abdomen can be achieved with cytoreductive surgery combined with hyperthermic intraperitoneal chemotherapy (HIPEC), definitive treatment of intrapleural progression may be of long-term benefit to the patient. Diseases where pleural extension of an intraabdominal disease has been reported include pseudomyxoma peritonei [1], peritoneal mesothelioma, and epithelial ovarian cancer. In these clinical situations, a patient must have disease control demonstrated within the abdomen and pelvis. Also, prior to the administration of intrapleural chemotherapy, a thoracic cytoreduction (pleurectomy and decortication) is performed in an attempt to remove all visible evidence of the malignant disease. In this paper, we report on the pharmacology of hyperthermic intrathoracic chemotherapy (HITOC) in patients with pseudomyxoma peritonei or peritoneal mesothelioma that had gained entrance to the chest through the hemidiaphragm. The chemotherapy agents studied are mitomycin C and doxorubicin. 

## 2. Materials and Methods

Permission to accumulate and analyze these data was obtained from the Ethics Committee at our institution. Patients undergoing pleurectomy and decortication followed by intrapleural chemotherapy for thoracic extension of pseudomyxoma peritonei and pleural mesothelioma were studied. These patients were identified through a prospective clinical database. The clinical management of these patients was as follows. Disease control within the abdomen and pelvis was determined over a minimum six-month time interval using abdominal and pelvic CT scans. Disease within the thoracic space was shown to progress over this time interval. After obtaining consent, the patients were taken to the operating room for a thoracotomy [2]. The extended right or left thoracotomy was accompanied by a resection of the seventh rib. First, a complete parietal peritonectomy was performed. Then, a partial visceral pleurectomy was performed, only removing pleura that was invaded by the malignant process. The visceral pleura within the pulmonary fissures was also carefully cytoreduced. Following completion of the cancer resection, the thoracic cavity was irrigated with copious warm saline solution and meticulous hemostasis obtained. The skin at the anterior and posterior extent of the thoracotomy incision was sutured shut with a running skin suture. The skin edges in the mid-portion of the chest cavity were elevated on a self-retaining retractor (Thompson Surgical Instruments, Traverse City, MI) in order to maintain a reservoir within the thoracic space. For infusion of chemotherapy solution, a Tenckhoff catheter was placed over the edge of the thoracotomy incision and secured by a suture. For drainage, a single 28 French straight thoracostomy tube was inserted through an intercostal space at the level of the hemidiaphragm posteriorly and directed up towards the apex of the chest. The chemotherapy solution was heated and repeatedly circulated by a hyperthermia pump (Belmont Instrument Corporation, Billerica, MA). Temperature of the chemotherapy solution was between 41 and 43°C within the hemithorax. During this dissection, the lung was maintained partially collapsed through the use of a double-lumen tube; the lung was allowed to inflate approximately half of its volume for the duration of the HITOC.

The patients received hyperthermic chemotherapy using mitomycin C at 15 mg/m^2^, doxorubicin at 15 mg/m^2^, and 5-fluorouracil at 400 mg/m^2^ with leucovorin at 20 mg/m^2^ given intravenously.

The concentration of mitomycin C and doxorubicin within the pleural fluid and plasma was determined at 15-minute intervals. HPLC assay was used to determine the concentration as described elsewhere [3, 4].

The carrier solution for the chemotherapy was 1.5% dextrose peritoneal dialysis solution. The chemotherapy was diluted in 2 liters of this carrier solution prior to instillation into the thoracic cavity through the Tenckhoff catheter.

All data presented on the graphs are +1 standard deviation. Calculations of area under the curve (AUC) and subsequent AUC ratios were obtained using GraphPad Prism analyses (GraphPad Software, Inc., La Jolla, CA).

For comparison of HITOC pharmacokinetics with hyperthermic intraperitoneal chemotherapy (HIPEC) pharmacokinetics, data from intraperitoneal and plasma drug concentrations in 25 consecutive patients were utilized. These patients were treated during the approximate time period as the patients receiving HITOC.

## 3. Results

**Figure 1:**
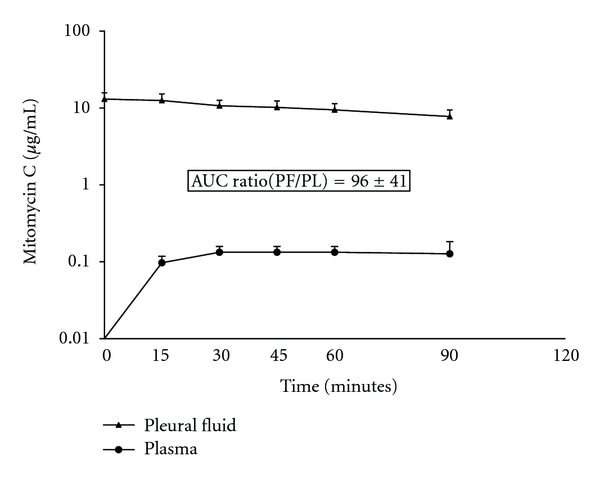
Pharmacokinetic study of mitomycin C instilled into the thoracic space following pleurectomy and decortication for pseudomyxoma peritonei spread by direct extension through the hemidiaphragm. The chemotherapy solution was maintained at 41–43°C by circulating the chemotherapy fluid through a hyperthermia pump.

For mitomycin C pharmacokinetics, three patients were available for study. All three patients had pseudomyxoma peritonei in the pleural space, and all three had a complete visible removal of disease at the time of thoracic cytoreduction. Two had a right thoracotomy and one had left-sided disease.  Figure 1 shows the area under the curve concentration times, time for the pleural fluid and for the plasma. The area under the curve pleural fluid to plasma ratio was 96 ± 41 over the 90 minutes. During the HITOC, 41 ± 3 percent of the total mitomycin C was absorbed from the thoracic space into the body compartment. 

**Figure 2:**
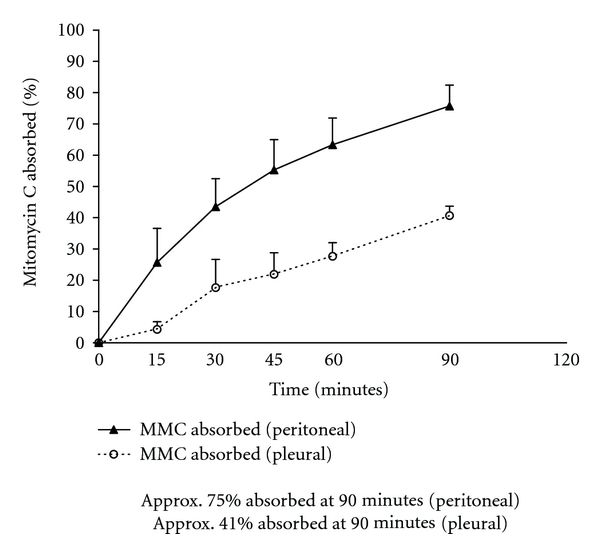
Percent mitomycin C absorbed from the chest cavity as compared to the peritoneal cavity following intrathoracic or intraperitoneal chemotherapy treatment. The chemotherapy solution in both groups of patients was maintained between 41 and 43°C by circulating the chemotherapy solution through a hyperthermia pump.

For comparison of HITOC mitomycin C clearance with HIPEC mitomycin C clearance, we used data from 25 patients whose pseudomyxoma was confined to the abdomen and pelvis. The mitomycin C was used at 15 mg/m^2^ and was diluted in 3 liters of 1.5% dextrose peritoneal dialysis solution. Temperature within the peritoneal space was 41 to 43°C.  Figure 2 shows the percentage of mitomycin C absorbed from the peritoneal space as compared to the pleural space. Approximately half of the amount of mitomycin C was absorbed from the pleural space as compared to the peritoneal space.

**Figure 3:**
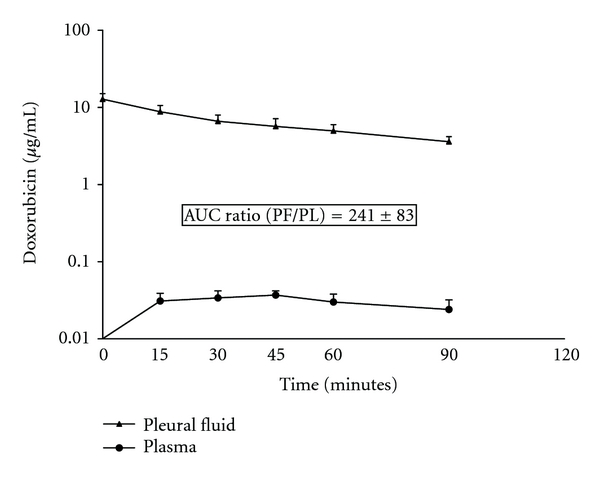
Pharmacokinetic study of doxorubicin in 4 patients who had drug instillation into the thoracic cavity. The chemotherapy solution was maintained between 41 and 43°C by circulation through a hyperthermia pump.

Figure 3 shows the area under the curve for pleural doxorubicin and for plasma doxorubicin in 4 patients treated with HITOC doxorubicin at 15 mg/m^2^ in 2 liters of 1.5% dextrose peritoneal dialysis solution. Two patients had pseudomyxoma peritonei and two had peritoneal mesothelioma. All patients had complete visible removal of disease at the time of thoracic cytoreduction. The area under the curve ratio was 241 ± 83.

**Figure 4:**
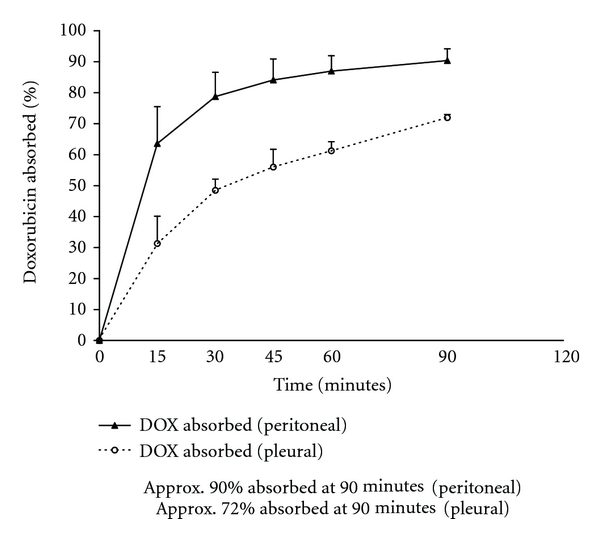
Percent doxorubicin absorbed from the pleural space as compared to the peritoneal cavity following intrathoracic or intraperitoneal chemotherapy treatment. The chemotherapy solution was maintained between 41 and 43°C by circulation through a hyperthermia pump.

In Figure 4, the percent of drug absorbed in 4 patients with HITOC with doxorubicin are compared to 25 patients who had HIPEC with doxorubicin. Seventy-two percent of this drug was absorbed at 90 minutes with intrapleural administration, and 90% was absorbed with intraperitoneal administration.

## 4. Discussion

Intrapleural chemotherapy continues to be well used palliatively in order to control debilitating and unrelenting pleural effusions from cancer. In this current application of intrapleural chemotherapy, the patient population was different in that no other known sites of disease were present in our patients. Therefore, the goal of the HITOC treatment was a curative one. The intrapleural chemotherapy administration was preceded by a thoracic cytoreduction of both parietal and involved visceral pleura. The goal of the cancer pleurectomy and decortication was to remove all visible evidence of disease. The role of the HITOC was to eliminate the microscopic residual disease that cannot be removed by cancer surgery. This strategy has been shown to be effective for peritoneal metastases from appendiceal malignancy, colorectal cancer, and peritoneal mesothelioma [5]. The rationale for this treatment within the chest cavity is the same as that within the peritoneal space.

### 4.1. Pharmacologic Advantage of Hyperthermic Intrathoracic Chemotherapy

Our data from patients treated with mitomycin C and with doxorubicin clearly show that the pharmacologic advantage of intracavitary chemotherapy exists within the thoracic space. This should result in a marked therapeutic benefit if the residual disease is of minimal extent so that the intrathoracic chemotherapy can penetrate the cancer cells. Entrance into the tissues surrounding the thoracic cavity is by simple diffusion [6]. An area under the curve ratio for mitomycin C of 96 ± 41 over the 90 minutes confirms the pharmacologic advantages of regional chemotherapy administration. Likewise, the area under the curve ratio of 241 for doxorubicin documents the same advantage.

### 4.2. Reduced Clearance of Intrapleural as Compared to Intraperitoneal Chemotherapy

In Figures 2 and 4, we calculated the percent of the total dose of cancer chemotherapy instilled at time 0 that was absorbed through the chest wall or through the partially deflated lung into the body compartment. The percent absorbed in 3 patients with HITOC mitomycin C was compared to 25 patients with HIPEC mitomycin C. Approximately half of the total quantity of mitomycin C instilled escaped from the pleural cavity as compared to the peritoneal cavity. Also, there was a reduction in the percent of doxorubicin absorbed from the pleural space as compared to the peritoneal space. Approximately 80% of the amount of drug was absorbed from the pleural space as compared to the peritoneal cavity.

In the two drugs used for HITOC in which we performed HPLC assays of chemotherapy concentration, both showed a high area under the curve ratio of pleural fluid to plasma. This reduced clearance from the pleural space resulted in a higher area under the curve ratio for intrapleural mitomycin C or doxorubicin as compared to intraperitoneal mitomycin C or doxorubicin. Our previous work with mitomycin C showed that the area under the curve ratio for intraabdominal treatment was approximately 27 [3]. It was almost three times as large in this study with intrapleural mitomycin C. The area under the curve ratio for intraperitoneal doxorubicin was approximately 79 [4]. Again, it was more than three times greater with intrapleural doxorubicin administration as compared to intrapleural instillation.

### 4.3. Speculations Regarding the Cause of Reduced Intrapleural Chemotherapy Clearance

The Dedrick model for predicting the clearance of intracavitary chemotherapy states that the permeability of the surface combined with the total diffusion surface controls the rate at which the concentration of a drug within the body cavity tends to normalize with that in the plasma [6]. A chest wall from which the pleura has been completely removed may be less permeable to mitomycin C and doxorubicin. Also, the partially deflated lung within the chest cavity filled by chemotherapy solution will be poorly perfused. Therefore, it may transmit drug less rapidly away from the lung surface. Most probably, the perfusion of the down lung is much less than the perfusion of the viscera absorbing and transporting drug within the abdomen and pelvis. Also, the pleural space generally has a capacity between 1 and 1.5 liters of chemotherapy solution. The intra-abdominal space has a 2-3 liter capacity for intraabdominal chemotherapy. This lesser volume of chemotherapy would result in a lesser total diffusion surface. 

### 4.4. Possible Need for Further Phase I/II Studies

Upon the initiation of our clinical experience with intrapleural chemotherapy, we considered an effort to increase the concentration of the intrapleural chemotherapy above that which has been used for many years within the abdominal space. However, two findings made it, we thought, unnecessary to perform further dose escalations. First of all, the control of pseudomyxoma peritonei and peritoneal mesothelioma in the chest cavity following thoracic cytoreduction and HITOC chemotherapy has approached 100%. Unpublished data shows 30 patients treated to date with no recurrences recorded. Also important regarding dose escalation is the morbidity and mortality seen with these studies. Complications resulting from parenchymal lung disease have been noted in 3 of the 30 patients (10%). Two patients developed pulmonary aspergillosis postoperatively and both of these patients went on to die. Another patient developed interstitial pneumonitis. This did not result in her demise but was a continuing problem in her limited survival. She died as a result of progressive disease within the abdomen. As a result of our clinical experience to date, further escalation of the intrathoracic chemotherapy concentration does not seem necessary. Our early clinical experience with thoracic cytoreduction and intrathoracic chemotherapy has been reported [1].

## References

[1] Pestieau SR, Esquivel J, Sugarbaker PH (2000). Pleural extension of mucinous tumor in patients with pseudomyxoma peritonei syndrome. *Annals of Surgical Oncology*.

[2] Alam NZ, Flores RJ, Rusch VW, Sugarbaker DJ, Bueno R, Krasna MJ, Mentzer SJ, Zellos L (2009). Pleurectomy and decortication for malignant pleural diseases. *Adult Chest Surgery*.

[3] van der Speeten K, Stuart OA, Chang D, Mahteme H, Sugarbaker PH (2011). Changes induced by surgical and clinical factors in the pharmacology of intraperitoneal mitomycin C in 145 patients with peritoneal carcinomatosis. *Cancer Chemotherapy and Pharmacology*.

[4] Sugarbaker PH, van der Speeten K, Stuart OA, Chang D (2011). Impact of surgical and clinical factors on the pharmacology of intraperitoneal doxorubicin in 145 patients with peritoneal carcinomatosis. *European Journal of Surgical Oncology*.

[5] Roviello F, Caruso S, Marrelli D (2011). Treatment of peritoneal carcinomatosis with cytoreductive surgery and hyperthermic intraperitoneal chemotherapy: state of the art and future developments. *Surgical Oncology*.

[6] Dedrick RL, Flessner MF (1997). Pharmacokinetic problems in peritoneal drug administration: tissue penetration and surface exposure. *Journal of the National Cancer Institute*.

